# Homemade pin-hook for surgical treatment of posterior cruciate ligament avulsion fractures

**DOI:** 10.1186/s12891-022-05892-8

**Published:** 2022-10-21

**Authors:** Qiang Guo, Xiaoning Li, Yifu Tang, Yuzhao Huang, Ling Luo

**Affiliations:** Department of Orthopaedics, The Third Xiangya Hospital, Central South University, 138 Tongzipo Road, Changsha, Hunan China

**Keywords:** Postero-medial approach, Homemade pin-hook, PCL tibial avulsion, Internal fixation

## Abstract

**Background:**

How to treat the posterior cruciate ligament (PCL) tibial insertion small and comminuted avulsion fracture is still challenging. Our study evaluated the clinical and radiological outcomes after ORIF of PCL tibial insertion avulsion fractures through the inverted L-shaped postero-medial approach using a homemade pin-hook.

**Methods:**

Between January 2009 and December 2020, twenty-four patients with isolated PCL tibial insertion bony avulsion were enrolled. There were 16 males and 8 females. The age range was 18-48 (32.5 ± 9.3) years. The time from injury to surgery was 1-10 (4.4 ± 2.8) days. There were 11 cases in the left knee and 13 cases in the right knee. The patients received anticoagulant therapy to prevent thrombosis. Preoperative standard X-ray, computerized tomography (CT) and magnetic resonance imaging (MRI) were performed. According to the Meyers-McKeever classification, there were 8 cases of type II and 16 cases of type III.

**Results:**

The operation time was 60-120 (89.6 ± 19.8) min. Postoperative follow-up ranged from 3 to 18 months. The average follow-up was 11.4 ± 4.3 months. In all patients, one or two homemade pin-hooks were used to fix different sizes of fracture segments. X-ray or CT scans taken after surgery revealed fracture union. The fractures healed in 9-16 (11.8 ± 1.7) weeks. At the last follow-up, the patients were able to fully straighten. The ROM (132.6° ± 3.9°), the Tegner-Lysholm score (96.2 ± 2.3) and the IKDC scores (95.5 ± 1.6) were all significantly improved compared with the preoperative values (77.5° ± 13.1°, 46.8 ± 8.9, 36.2 ± 7.9). The posterior drawer test was negative. The gastrocnemius muscle strength did not diminish. No internal fixation migration was observed during the follow-up. No neurovascular bundle- or hardware-related complications were reported.

**Conclusions:**

The inverted L-shaped postero-medial approach with homemade pin-hook fixation for the treatment of PCL avulsion fractures produces acceptable clinical and radiological results. Moreover, the homemade pin-hook made of K-wires is affordable and reduces patient costs. It is a practical application and worth recommending, especially for community hospitals.

## Background

The function of a strong posterior cruciate ligament (PCL) is to prevent posterior tibial translation, especially in the knee flexion position. PCL avulsion fractures, representing approximately 20% of ligamentous knee injuries, cause knee instability. If not treated, such fractures can lead to accelerating articular degenerative changes due to increased pathological articular contact forces [1]. Surgery for Meyers-McKeever type II and III fractures is considered necessary to achieve anatomic reduction and obtain a stable knee [2].

Currently, the posterior approach and arthroscopic management are commonly described methods [3]. The disadvantages of the traditional posterior approach include extensive soft exposure and a high risk of iatrogenic neurovascular impairment [4]. Modified posterior approaches, such as oblique incision and inverted L-shaped postero-medial approaches, have been introduced with minimal invasiveness and satisfactory follow-up outcomes [2, 5]. Arthroscopic treatment for PCL bony avulsion fractures is technically difficult and probably beyond the capabilities of non–sports-fellowship-trained orthopaedists. In the present study, the inverted L-shaped postero-medial approach was employed.

**Fig. 1:**
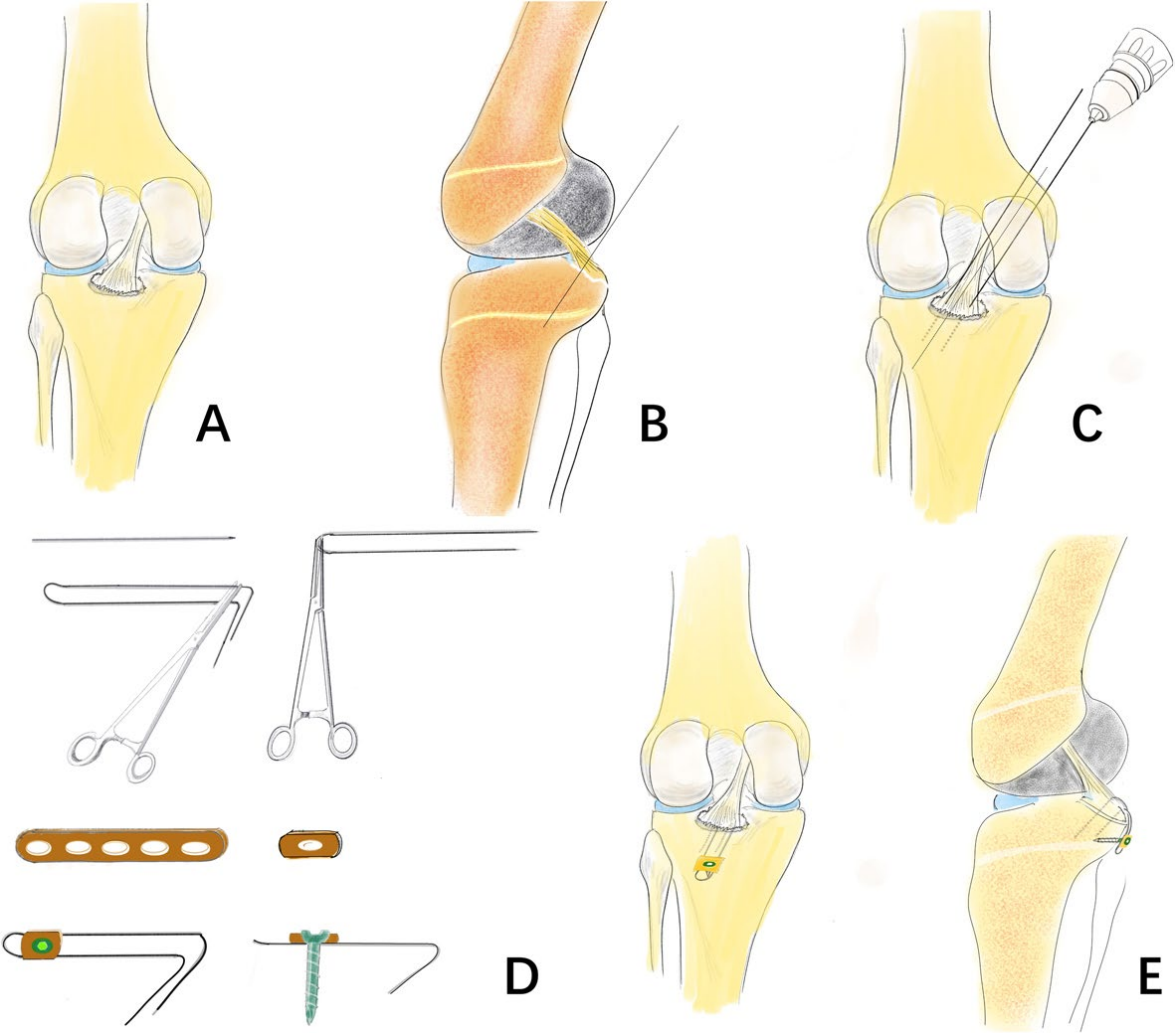
The schematic diagram of pin-hook fixation of posterior cruciate ligament avulsion fractures. **A** The exposure of PCL avulsion fracture fragments; **B** The reduction and temporary fixation of PCL avulsion fracture fragments; **C** Paving the path for the pin-hook(the diameter of the drilling K-wire is 0.5 mm larger than that of the pin-hook); **D** The production process of pin-hook system; **E** The fixation of posterior cruciate ligament avulsion fractures using pin-hook system

**Fig. 2:**
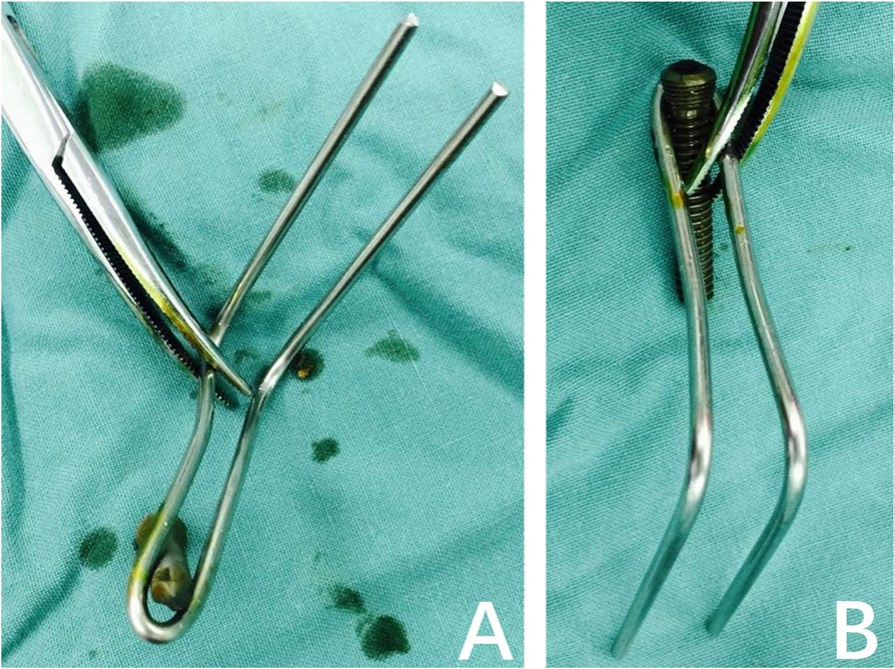
Shape of the innovative homemade pin-hook system made with Kirschner wire and screw

The methods used to repair PCL avulsion fracture are determined by the fracture segment size, as well as the fragment’s comminution. These injuries generally occur at the PCL tibial attachment and may involve either a small area at the connection’s posterior region or a large area that extends anteriorly. Internal fixation materials now available include metal screws, plates and screws, toothed plates combined with hollow lag screws and sutures [6]. However, there is no standard implant, successful repair of tiny fractures remains difficult, and the related research is still limited. We built a Kirschner wire into a unique frame called a pin-hook (Figs. 1D, 2A-B) for PCL avulsion fracture fixation. The present study is the first to assess the clinical and functional outcomes of our innovative homemade pin-hook for the surgical treatment of isolated posterior cruciate ligament avulsion fractures by an inverted L-shaped postero-medial approach.

## Methods

### Patients

Patients without previous knee injury who were diagnosed with isolated acute Meyers-McKeever type II or III PCL tibial avulsion fractures were enrolled, and the follow-up records were obtained. Patients with one of the following conditions were excluded: 1) any other associated bony or other ligament injuries; 2) neurovascular injury.

**Fig. 3:**
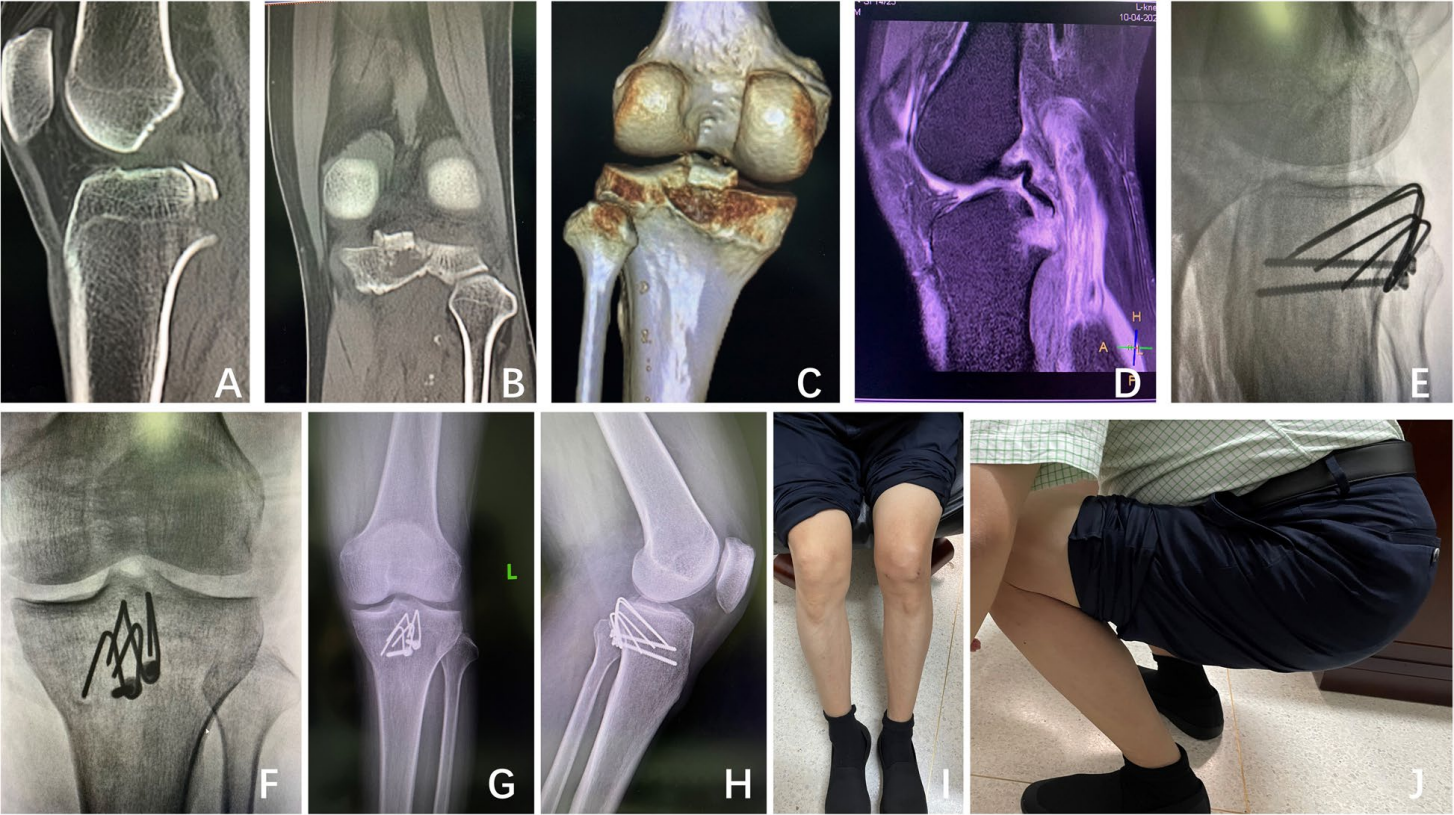
The case I was diagnosed with an avulsed left PCL tibial insertion caused by traffic accidents. **A**-**D** Preoperative CT scan and MRI confirming the PCL avulsion fracture; **E**-**F** Postoperative fluoroscopy showing the optimum position of the homemade pin-hook and fracture reduction; **G**-**H** Three-month postoperative follow-up radiographs showing fracture union and no loosening of internal fixation. **I**-**J** Three-month postoperative photos displaying good knee function without any discomfort

**Fig. 4:**
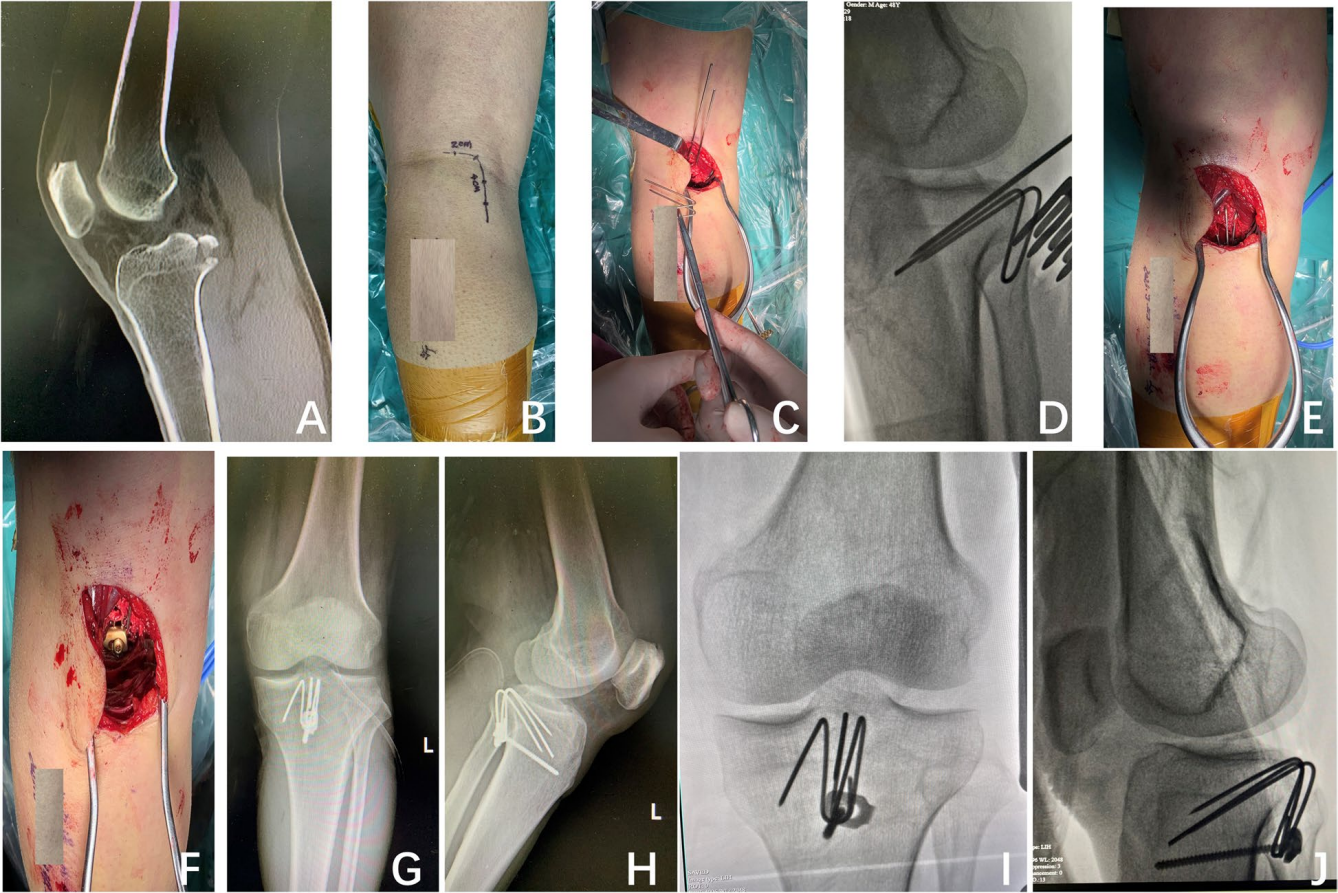
The case II presented with an acute knee injury, diagnosed as an avulsed left PCL tibial insertion. **A** Preoperative CT scan confirming the PCL avulsion fracture; **B** The planned inverted L-shaped postero-medial skin incision; **C** The first homemade pin-hook was implanted; **D**-**E** The second homemade pin-hook was implanted; **F** The pin-hook was fixed with a screw and a one-hole reconstruction plate; **G**-**H** Postoperative fluoroscopy showing the optimum position of the homemade pin-hook and fracture reduction; **I**-**J** Three-month postoperative follow-up radiographs showing fracture union and optimum position of internal fixation

This was a retrospective case series. Between January 2009 and December 2020, twenty-four patients meeting the inclusion criteria were enrolled. The surgeries were performed by a single surgeon (L.L.). There were eight females and sixteen males. The age distribution was 18-48 (32.5 ± 9.3) years. The time from injury to surgery was 1-10 (4.4 ± 2.8) days. There were 11 cases in the left knee and 13 cases in the right knee. The patients received anticoagulant therapy to prevent thrombosis. Preoperative standard X-ray, computerized tomography (CT) scan and magnetic resonance imaging (MRI) were performed (Figs. 3A-D and 4A). There were 8 cases of Meyers-McKeever type II and 16 cases of Meyers-McKeever type III. Knee stability was clinically assessed again under anaesthesia. The clinical details of the patients are presented in Table 1.

**Table 1:** Patients demographics and clinical indicators

Age (mean，range，yr)	32.5 ± 9.3 years(18 to 48)
Sex(m/f，%)	16 males (66.7%)
	8 females (33.3%)
Surgery time (mean, range, day)	4.4 ± 2.8 days(1 to 10)
Injury position(l/r,%)	11 left (45.8%)
	13 right (54.2%)
Meyers-McKeever classification(n,%)	8 type II (33.3%)
	16 type III (66.7%)
Operation time (mean, range, min)	89.6 ± 19.8 mins (60-120)
Follow up (mean, range, month)	11.4 ± 4.3 months (3-18)
Fracture healing time (mean, range, week)	11.8 ± 1.7 weeks (9-16)

### Surgical methods

All patients were treated with a homemade pin-hook by the inverted L-shaped postero-medial approach. After induction of anaesthesia, the patient was positioned prone on a radiolucent table with a pneumatic tourniquet. The knee was slightly flexed. A 6-8 cm inverted L-shaped incision was made on the postero-medial side of the popliteal fossa (Fig. 4B). The incision was made along with the transverse skin striation of the popliteal fossa and extended to the medial border of the gastrocnemius muscle. Then, it turned at the medial corner of the popliteal fossa and reached the distal end. The medial head of the gastrocnemius muscle and semimembranous muscle were visualized with blunt dissections along the intermuscular gap and then retracted laterally, protecting the neurovascular bundle and showing the back of the knee capsule. The joint capsule was cut longitudinally to reveal the PCL avulsion fracture fragment (Fig. 1A). Haematoma and soft tissue that extruded into the fracture area were removed. The avulsion fracture was reduced to its bed under direct vision with the knee flexed approximately 30°. The K-wires were used to fix it provisionally (Figs. 1B and 4C-D). Then, the quality of reduction was checked by fluoroscopy.

Taking case II as an example, two 2.0 mm Kirschner wires were used to pave the path for the pin-hook. Generally, the diameter of the drilling K-wire was 0.5 mm larger than that of the pin-hook (Figs. 1C and 4C-D). Two 1.5 mm Kirschner wires were utilized to form a pin-hook with one sharp tooth hook and two sharp tooth hooks (Figs. 1D and 4E-F). In clinical practice, the size and radian of the pin-hook, as well as the distances between the hooks, are dependent on the bone fragments to provide stability. The hooks were inserted along with the drilling hole to grasp the fracture segments. Then, the second one sharp tooth pin-hook was implanted in a similar way. The distal end of the two pin-hooks was both compressed to the bone surface by a 3.5 mm cortical screw with one-hole reconstruction plate (Figs. 1D-E and 4E-J). Sometimes, the screws only could be applied in case I (Figs. 2A-B and 3E-J). The location of the implant and the length of the screw were confirmed under C-arm fluoroscopy when the knee was kept in flexion or extension to ensure the stability of fixation. The joint capsule was closed, and a drainage tube was installed. The subcutaneous tissue and skin were sutured.

### Postoperative treatment

Our postoperative care included managing moderate to severe pain. The drainage tube was removed 24 h postoperatively. X-ray and CT were performed again to confirm the reduction and implant position. Sequential rehabilitation included quadriceps strengthening and range of motion training by the time of drain removal, a gradual increase in weight-bearing from toe-touch weight-bearing with crutches 4 weeks after surgery, and full weight-bearing 8 weeks after surgery. Other activities were recommended after half a year.

### Clinical indicators

Operation time, blood loss and complications were documented. X-ray and CT scans were taken to monitor fracture healing. The posterior drawer test was used to assess knee joint laxity. It was classified as positive or negative referring to the contralateral knee. The range of motion (ROM) was measured using a conventional goniometer. The International Knee Documentation Committee (IKDC) and the Tegner-Lysholm score were used to assess the function of the knee joint. The heel raise test was carried out using a single-leg stance to evaluate gastrocnemius muscle strength.

### Statistical processing

SPSS 20.0 statistical software (IBM Corp., Armonk, NY) was applied to analyse the data. Data with a normal distribution are expressed as the mean ± standard deviation and analyzed by T-test or one-way ANOVA. Data with a nonnormal distribution are expressed as the median (P_25_, P_75_) and analyzed by Mann-Whitney U test or Kruskal Wallis H test. Counting data are expressed as the rate (%). A *P* < 0.05 was considered statistical significance.

## Results

All patients’ incisions healed. There were no complications, such as bone nonunion, infection, deep-vein thrombosis, or atypical haematomas. The operation time was 60-120 (89.6 ± 19.8) min. The average follow-up was 11.4 ± 4.3 months, ranging from 3 to 18 months. X-ray and CT scans revealed that the fractures had healed. The fractures healed in 9-16 (11.8 ± 1.7) weeks. At the last follow-up, the ROM(132.6° ± 3.9°), the Tegner-Lysholm score (96.2 ± 2.3) and the IKDC scores(95.5 ± 1.6) were all significantly improved compared with the preoperative values (77.5° ± 13.1°, 46.8 ± 8.9, 36.2 ± 7.9). The posterior drawer test was negative. The gastrocnemius muscle strength did not diminish. No internal fixation migration was observed during the follow-up. No hardware-related complications were reported during the follow-up (Table 2).

**Table 2:** Outcomes at the final follow-up

	Preoperative	Final follow-up	*P* value
ROM	77.5° ± 13.1°	132.6° ± 3.9°	<0.001
Tagner-Lysholm score	46.8 ± 8.9	96.2 ± 2.3	<0.001
IKDC	36.2 ± 7.9	95.5 ± 1.6	<0.001

## Discussion

Avulsion injuries of the PCL, although rare, can lead to significant morbidity when not recognized and treated properly [7, 8]. The most common mechanism in car traffic accidents is dashboard or chair back collision, in which a direct force from hard braking is applied to the proximal part of the tibia in an anterior-to-posterior direction, with the knee in flexion [9]. The knee morphology, such as a smaller notch width index (coronal), was found to affect PCL avulsion fracture in women but not in men [10]. If left untreated, the injury leads to secondary joint changes resulting in osteoarthritis. According to the commonly used Meyers-McKeever system, conservative therapy can yield good outcomes in individuals with Meyers-McKeever type I fractures, but type II and III fractures are typically treated surgically. Some scholars conclude that acute PCL avulsion injuries with displacements of less than 6.7 mm should be considered for nonoperative treatment [11]. Other researchers found that surgery can help restore the tension of the PCL and obtain a better prognosis than nonsurgical treatment. Surgery also contributes to early activity and reduces complications of long-term breakage, such as deep vein thrombosis and stiff knees.

With the advancement of less-invasive arthroscopic technology, arthroscopic surgery for the treatment of PCL avulsion fractures has become increasingly popular [12–14]. Meanwhile, it can identify damage to other structures of the knee [15]. Both arthroscopic and open methods for the treatment of PCL tibial-side avulsion injuries result in comparably good clinical outcomes, radiological healing, and stable knees according to a systematic review [16]. It has been observed that anatomical reduction is difficult to achieve by arthroscopy-assisted internal fixation for PCL avulsion fractures. Despite being less invasive and capable of managing concurrent knee problems, arthroscopic surgery has disadvantages of being technically difficult, requiring more operating time, necessitating specific equipment and preparations, and having a long learning curve. Furthermore, the price is relatively high. Thus, it is not ideal for low-income communities where arthroscopic surgery is not available.

A posterior median S-shaped approach is required to cut off part of the medial head of the gastrocnemius muscle, which leads to muscular weakening after surgery. This obviously increases the risk of vascular and nerve damage when exposing the PCL tibial stop. The patients in this study were treated with a postero-medial arc incision. The medial head of the gastrocnemius muscle is pulled laterally from the gap between the medial head of the gastrocnemius muscle and semimembranous muscle, which aids in the effective protection of vascular and nerve bundles. This incision is simple with little scarring. Otherwise, a large scar across the transverse skin striation of the popliteal fossa will influence knee extension. The way of exposing the fracture site in this study had little effect on postoperative gastrocnemius weakening. The avulsion fracture was under direct view throughout the procedure, making it easy to carry out fracture reduction. In addition, this helped prevent re-embedding of the surrounding soft tissue into the fracture site during reduction and decreased the likelihood of fracture nonunion.

Debate still exists regarding the technique for fixation [17, 18]. Perfect fixation should have the advantages of easy operation, reliable reduction and appropriateness for popularization. Metal screws [19], plates [20], toothed plates combined with hollow lag screws [2] and sutures [15, 21] are surgical fixation materials reported in the literature. Screw fixation is a reliable technique for major avulsion fractures, but it can induce refragmentation and unstable fixation in minor avulsed or comminuted fractures. A screw cannot fix an avulsed fragment that is too small (< 20 mm^2^) [22]. Sutures are linked with bone cutting and fixation loosening and do not allow early functional exercise [23]. Although these techniques have produced good results in certain studies, problems such as implant discomfort prompting implant removal, fragmentation of the PCL and, more significantly, loss of fixation with additional revision have been noted.

Our innovative homemade pin-hook is flexible for use in large bony fractures or tiny comminuted fractures, can provide firm fixation with the help of tooth-like hooks and has little effect on knee function (Figs. 3G-J and 4G-J). Cutting damage is minimized compared to that with other implants. Our pin-hook is made of Kirschner wire, which is cheaper than other materials. The procedure does not require additional surgical skills and is very easy for surgeons to master. Moreover, use of the homemade pin-hook is feasible even in underdeveloped places. The special characteristics of the pin-hook control fracture rotation. The remote screws are placed in typical cortical bone to prevent fixation loosening or withdrawal during knee activities. In the present study, all the fractures healed within 4 months following surgery due to the excellent reduction of the fracture under direct vision. The homemade pin-hook made of Kirschner wire also reduces the risk of infection and internal fixation-related complications. It can also be applied for the treatment of open posterior cruciate ligament avulsion fractures.

There are some limitations to our investigation. Further randomized well-controlled comparative studies with a larger sample size are needed to corroborate the current study’s findings, and a biomechanical investigation is required to evaluate different fixation tools and to determine the superiority of the pin-hook. As the Kirschner pin-hook will influence MRI examinations, the exploration of new materials is necessary.

## Conclusions

The inverted L-shaped postero-medial approach with homemade pin- hook fixation in the treatment of PCL avulsion fractures produces acceptable clinical and radiological results. Moreover, the homemade pin- hook made of K-wires is affordable and reduces patient costs. It is a practical application and worth recommending, especially for community hospitals.

## Data Availability

The datasets used and/or analysed during the current study are available from the corresponding author on reasonable request.
